# A cross-sectional testing of The Iowa Personality Disorder Screen in a psychiatric outpatient setting

**DOI:** 10.1186/1471-244X-11-105

**Published:** 2011-06-28

**Authors:** Ingrid Olssøn, Øystein Sørebø, Alv A Dahl

**Affiliations:** 1 Department of Psychiatry, Innlandet Hospital Trust, N-2318 Hamar, Norway; 2 Schools of Business and Social Sciences, Buskerud University College, N-3511 Hønefoss, Norway; 3 Department of Oncology, Oslo University Hospital and University of Oslo, N-0310 Oslo, Norway

**Keywords:** Personality disorders, Screening instrument, Iowa Personality Disorder Screen, Psychometrics

## Abstract

**Background:**

Patients suspected of personality disorders (PDs) by general practitioners are frequently referred to psychiatric outpatient clinics (POCs). In that setting an effective screening instrument for PDs would be helpful due to resource constraints. This study evaluates the properties of The Iowa Personality Disorder Screen (IPDS) as a screening instrument for PDs at a POC.

**Methods:**

In a cross-sectional design 145 patients filled in the IPDS and were examined with the SCID-II interview as reference. Various case-findings properties were tested, interference of socio-demographic and other psychopathology were investigated by logistic regression and relationships of the IPDS and the concept of PDs were studied by a latent variable path analysis.

**Results:**

We found that socio-demographic and psychopathological factors hardly disturbed the IPDS as screening instrument. With a cut-off ≥4 the 11 items IPDS version had sensitivity 0.77 and specificity 0.71. A brief 5 items version showed sensitivity 0.82 and specificity 0.74 with cut-off ≥ 2. With exception for one item, the IPDS variables loaded adequately on their respective first order variables, and the five first order variables loaded in general adequately on their second order variable.

**Conclusion:**

Our results support the IPDS as a useful screening instrument for PDs present or absent in the POC setting.

## Background

Several studies have indicated that the prevalence of personality disorders (PDs) is high in the setting of psychiatric outpatient clinics (POCs). From the United States Zimmerman reported a prevalence of 50% [1], while 80% was found by Alnæs & Torgersen [2] in Norway. The variation in prevalence rate depends in part on practical matters like the referral practice of the general practitioners (GPs), and in part on research matters like the instruments used to assess PDs. Frequent co-morbidity of Axis I disorders and PDs regularly demands extensive diagnostic assessments [3,4], and PD as an influential but unacknowledged factor impedes the referral process [5]. The GPs want a qualified diagnostic assessment and advice for further treatment as feedback of their referrals. A correct diagnosis of PDs is of clinical importance since their presence is associated with longer duration, poorer treatment outcome and recurrence of Axis I disorders [6-8]. Identification of such co-morbidity is therefore also important for the choice of treatment [9,10]. All these issues make diagnostic evaluation of PDs an important matter at POCs.

Structured interviews are considered as the most reliable and valid method for the diagnostic assessment of PDs [11], but they are time-consuming and demand substantial clinical competence of the interviewer. At POCs in Norway, such clinical competence is a limited resource and the pressure to evaluate patients is considerable, and for efficient and qualified diagnostic assessment of PD a psychometrically valid screening instrument for PDs would be very helpful in the POC setting.

The Iowa Personality Disorder Screen (IPDS) is an 11 items interview-based screening instrument for identification if PD is present or absent, using diagnoses based on the Structured Interview for DSM-III-R Personality Disorders (SIDP-R) as reference [12]. The authors also tested different subsets of five to seven items in order to identify the presence of PDs. An optimal sensitivity of 92% and specificity of 79% were observed for the IPDS in their clinical sample with a PDs base rate of 46%. In a replication study, Trull et al. [13] reported an optimal sensitivity of 69% and a specificity of 91% for the IPDS in a non-clinical sample with a PDs base rate of 35%. The screening properties of a self-report version of the IPDS were evaluated by Morse et al. [14]. They found the optimal sensitivity of 80% and specificity of 55% in their sub-sample of psychiatric patients with a base rate of 84% PDs, and somewhat poorer values in their non-psychiatric subsample with a base rate of 44%. Recently, Germans et al. [15] tested the IPDS self report version with the The Structured Clinical Interview for DSM-IV Axis II Disorder (SCID-II) [16] as reference in a Dutch sample of psychiatric outpatients (N = 195) with a base rate of 50% PDs. They reported an optimal sensitivity of 77% and specificity of 85%. The IPDS was based on eleven diagnostic criteria defined by DSM-III-R, and ten of these items were retained in DSM-IV.

In the four studies of the IPDS published so far, the sensitivity and specificity of the IPDS have shown somewhat variable results. This may be due to differences between the interview and self-rating formats, as well as small sample sizes and variable base rates of PDs. In this study from Hamar POC, we tested the IPDS self-rated version with the SCID-II as reference (gold standard). We investigated three research questions: 1) Do socio-demographic and other psychopathology influence the screening properties of the IPDS? 2) What are the sensitivities and specificities of the IPDS items alone and in combination, and 3) What are the relationships of the 11 IPDS items and the concept of PDs as studied by latent variable path analysis?

## Methods

### Material

Exclusion criteria for the study were age < 20 years, clinically assessed cognitive impairment, psychosis, severe somatic illness, or problems regarding Norwegian language. Due to the organisation of the POC patients referred with alcohol or drug dependence as main diagnoses were excluded, while abuse diagnoses were accepted for inclusion. Suicidality was assessed by the clinical interviews and patients with severe suicidality in need of immediate hospitalization were excluded, while lower levels of suicidality were not defined as an exclusion criterion. The therapists asked their eligible patients if they were willing to participate in the study. A stratified recruitment procedure was used in order to get a sample of 50% patients diagnosed with PDs and 50% without. From the start of inclusion February 1, 2009 we included both types of patients, however, when the proportion of non-PD patients was filled, only PDs patients were included. The inclusion period ended on May 15, 2010.

### Procedure

The IPDS was part of a questionnaire filled in by the patients after they had given informed consent. The SCID-II interviews were done by their therapists who were blind to the patients' questionnaire ratings. The time between the IPDS self-rating and the SCID-II interview varied from 3 days to eight weeks. Preceding the inclusion period, the experienced therapists participated in a two day intensive SCID-II seminar covering theoretical aspects, scoring of video interviews with discussions, organized by experts from the Department for Personality Psychiatry at Ullevaal University Hospital, Oslo.

### Measures

#### Self-rated measures

*The IPDS *contains 11 items which correspond to diagnostic criteria for PDs which showed the best discriminative ability in the study by Langbehn et al. [12]. These items are scored "yes" (1) or "no" (0), and an IPDS sum score ranging from 0 to 11 is calculated. When rating the items, the patients are instructed to look back to their usually self if the ways they have been in recent weeks or months are different from the way they usually are. The IPDS was translated and back-translated into Norwegian by the last author with permission from Bruce Pfohl, MD. Adaption of the IPDS into a self-administered questionnaire did not require any special procedure. The items are given in Table 1 with their location in DSM-IV.

**Table 1:** Item endorsement, internal consistency, sensitivity and specificity of the 11 items of the IPDS

Item (personality disorder criterion number in DSM-IV)	Frequency (%) (N = 145)	Internal consistency^a^	Sensitivity	Specificity	PVP ^b^	PVN ^c^	CC^d^
1. Marked shift in mood (BRD-6)	39	0.69	0.77	0.56	0.64	0.70	0.66
2. Uncomfortable without attention (HST-2)	3	0.72	0.06	0.99	0.80	0.99	0.52
3. Actions to obtain immediate satisfaction (HST)*	23	0.72	0.29	0.82	0.03	0.53	0.55
4. Reluctant to confine in others (PAR-3)	42	0.69	0.60	0.75	0.70	0.64	0.67
5. Excessive social anxiety (AVD-1/5)	53	0.68	0.81	0.75	0.77	0.79	0.78
6. Unwilling to get involved unless liked (AVD-2)	49	0.69	0.73	0.75	0.75	0.73	0.74
7. Lack of stable self-image (BRD-3)	23	0.69	0.38	0.92	0.82	0.59	0.65
8. Prone to overemphasis importance (NAR-2/3)	25	0.71	0.33	0.83	0.67	0.55	0.58
9. Expects to be exploited or harmed (PAR-1)	34	0.68	0.52	0.85	0.78	0.64	0.68
10. Bear grudges or is unforgiving (PAR-5)	55	0.72	0.67	0.56	0.61	0.63	0.62
11. Insensitive to others concerns and needs (NAR-2/3)	22	0.71	0.33	0.89	0.75	0.57	0.61

^a ^α coefficient if item deleted, overall α coefficient is 0.72. ^b ^PVP: Predictive value of a positive test. ^c ^PVN: Predictive value of a negative test ^d ^Correctly classified. * Histrionic PD criterion 7 in DSM-III-R was not retained in DSM-IV

BRD: Borderline PD; HST: Histrionic PD, PAR: Paranoid PD; AVD: Avoidant PD, NAR: Narcissistic PD.

*The Global Severity Index (GSI) *is derived from the The Symptom Check-List 90 Revised [17] based on The Hopkins Symptom Checklist [18], and reflects the general symptom level of the individual in the previous seven days. The SCL-90R consists of 90 items which are rated on a five-point Likert scale (0-4) from "not at all" to "extremely". The GSI is the sum of the item scores divided with 90, and a GSI score of ≥0.85 (males) and ≥0.70 (females) separates individuals with caseness of mental distress from those without [19].

*Socio-demographic variables: Relationship status *was dichotomized into paired and non-paired, and *basic level of education *was divided into ≤12 years of education (low level) and >12 years (high level). *Work status *was classified into 'paid work' versus 'not in paid work'. Those who were employed full time, part time or were self-employed belonged to the former category, while others (i.e. unemployed, retired or on disability pension) belonged to the latter. We included two items from the Health Survey of Nord-Trøndelag County (http://www.ntnu.no/hunt/skjema). *Self-rated health *was rated by the item: "How is your current health?" with a four point Likert-scale ('bad'/'not so good'/'good'/'very good'), which was dichotomized into "good health" and "poor health" with two scale scores in each categories. *General satisfaction with life *was rated on a seven point Likert-scale from one ('very satisfied') to seven ('very dissatisfied') and in the analyses dichotomised into "satisfied" (1-3) and "dissatisfied" (4-7).

#### Interview-based measures

*The SCID-II *diagnoses of PDs were the diagnostic references in this study. The SCID-II is a semi structured interview for the assessment of PDs according to DSM IV [20] and covers ten different PDs and in addition PD not otherwise specified (PD-NOS) [16]. We diagnosed PD-NOS if the therapist scored nine or more positive criteria on the SCID-II without reaching the threshold for any specific PDs. We used the official Norwegian SCID-II, revised version 2004.

*The MINI International Neuropsychiatric Interview (MINI) *is a brief structured diagnostic interview for Axis I diagnoses. The reliability and the validity of the MINI are considered to be good [21]. In this study we used the Norwegian version 5.0.0 of the MINI, revised 2007.

*Global Assessment of Functioning (GAF) *[20] is a commonly used rating scale for assessing patients' overall mental health reflecting psychological, social and occupational functioning. The GAF-Split version was used in this study, assessing symptom and function scores separately [22].

### Statistics

The statistics analyses were performed with SPSS for Windows, version 17.0 and Partial Least Squares Path Modeling (PLSPM) with XlStat version 2010.2.03. The internal consistency of the IPDS was evaluated by Cronbach's coefficient alpha. The receiver operating curve for the IPDS score versus PDs present or absent was produced, and the area under the curve was calculated. We tested if other variables interfered with the associations between the IPDS score as independent variable and PDs present or absent as dependent variable using bivariate and multivariate logistic regression analyses. The strength of the associations was expressed as odds ratios (ORs) with 95% confidence intervals.

**Figure 1:**
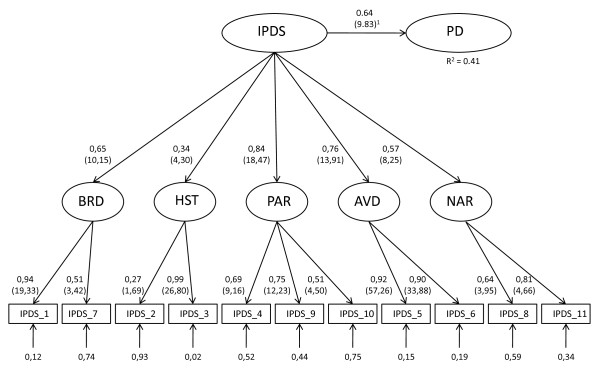
PLS Path Model with the IPDS as second orders construct that explains PDs*. *Explanation of abbreviations: PD: personality disorders; IPDS: The Iowa Personality Disorders Screen; BRD: Borderline PD; HST: Histrionic PD, PAR: Paranoid PD; AVD: Avoidant PD, NAR: Narcissistic PD. *Explanation of statistics*: All numbers in parentheses are t-values (>1.96 = p ≤ 0.05). The number 0.64 above the line between IPDS and PD is a standardized regression coefficient and 0.64^2 ^indicates how much IPDS explains of the variance in PD (i.e. 41%). The eleven numbers at the bottom of Figure 1 (i.e. without corresponding parentheses) indicates the amount of measurement error in each IPDS-item. The remaining numbers in Figure 1 represents second and first order factor loadings.

We constructed a hierarchical IPDS model consisting of the measured IPDS items, a set of identified first order latent variables and the IPDS as a second order variable using the key steps in PLSPM recommended by Wetzels et al. [23]. In turn the second order IPDS variable, based on the hierarchical IPDS specification, was specified as an exogenous variable in a model with PD as the endogenous variable (cf. Figure 1). In the evaluation of the PLSPM model, a t-value higher or equal to 1.96 represents significant findings (p ≤ 0.05). Hence, the significance level was set at p ≤ 0.05, and all tests beyond the PLSPM were two-sided.

### Ethics

The study was approved by The National Committee for Research Ethics of Health Region South-East. All participants gave written informed consent.

## Results

### Sample description

In total 156 interviews and self-ratings were completed. Individuals with attention deficit/conduct disorders were excluded (N = 11) due to lack of sufficient concentration for completion of the SCID-II interview and the questionnaire. The study sample therefore consisted of 145 patients, 61% (N = 89) women and 39% men, with mean age 37.8 (SD 11.8) years.

Based on the SCID-II interview 73 patients had a total of 95 PDs, mainly belonging to cluster C (51% of the PDs diagnoses) with 18% of diagnoses in cluster A, 14% cluster B, and 18% PD-NOS (Table 2). Concerning Axis I disorders based on the MINI, mood disorders were most common (72%, N = 105) followed by anxiety disorders (23%, N = 33) (Table 3). More than one Axis I disorder was found in 43% (N = 63) of the patients.

**Table 2:** Number of patients with one or more PDs according to the SCID-II and the IPDS

Personality disorders	SCID-II	IPDS*	
	N = 73	Hit rate	Non-hit rate
*Cluster A*			
Paranoid	16	13/16	3/16
Schizotypal	0	-	-
Schizoid	1	0/1	1/1
Total cluster A	17	13/17	4/17
*Cluster B*			
Histrionic	0	-	-
Narcissistic	1	1/1	0/1
Borderline	10	8/10	2/10
Antisocial	2	1/2	1/2
Total cluster B	13	10/13	3/13
*Cluster C*			
Avoidant	40	32/40	8/40
Dependent	2	2/2	0/40
Obsessive-compulsive	6	6/6	0/6
Total cluster C	48	40/48	8/48
Personality disorder NOS	17	16/17	1/17
Personality disorders total	95	79/95 (83%)	16/95 (17%)

* Cut-off level ≥4 of 11 item version

**Table 3:** Logistic regression analyses of various independent variables and SCID-II personality disorder present or absent as dependent variable (N = 145)

Variables	Sample	Bivariate analysis			Multivariate analysis		
	N = 145 (%)	OR	95%CI	P	OR	95%CI	P
IPDS sum score		2.14	1.67 - 2.73	<0.001	2.12	1.66 - 2.97	<0.001
*Gender*		0.98	0.50 - 1.51	0.95			
Female	89 (61)						
Male	56 (39)						
*Relationship status*		0.95	0.49 - 1.85	0.88			
Paired relation	62 (44)						
Non-paired	80 (56)						
*Level of education*		1.72	0.88 - 3.35	0.11			
> 12 years	59 (41)						
≤ 12 years	86 (59)						
*Work status*		1.87	0.94 - 3.73	0.07	1.21	0.49 - 3.00	0.68
Paid work	53 (36)						
Not in paid work	92 (63)						
*Self-rated health*		1.94	0.88 - 4.26	0.1	1.35	0.45 - 4.04	0.6
Good health	34 (24)						
Poor health	110 (76)						
*General satisfaction*		1.43	0.64 - 3.22	0.39			
Satisfied	30 (21)						
Dissatisfied	113 (79)						
*Comorbid Axis I disorders*							
Mood disorders	105 (72)	1.02	0.47 - 2.14	0.96			
Anxiety disorders	33 (23)	1.06	0.49-2.31	0.88			
	**Mean (SD)**						
*Age *	37.8 (11.8)	0.99	0.96 - 1.02	0.35			
*GSI *	1.5 (0.7)	3.54	1.95 - 6.42	<0.001	0.68	0.29 - 1.62	0.34
GAF S*	55 (7)	0.93	0.88 - 0.98	<0.001	0.93	0.86 - 1.0	0.06
*GAF F*	55 (9)	0.94	0.90 - 0.98	<0.001	-	-	-

* Correlation between GAF-S and GAF-F is 0.70, so only GAF-S was entered into the multivariate analysis.

### Factors associated with PDs diagnoses

In bivariate analyses the IPDS score was significantly associated with PDs present or absent, but so was also the GSI, GAF-S and GAF-F scores (Table 3). In multivariate analysis only the IPDS score showed a persistent significant association with PDs.

### IPDS item description

The prevalence of positive criteria varied from 3% of the patients (IPDS-2) to 55% (IPDS-10) (Table 1). The internal consistency of the IPDS was Cronbach's coefficient alpha 0.72, and the alpha values when one item was omitted varied between 0.68 and 0.72.

The IPDS-5 showed the highest sensitivity 0.81, while IPDS-2 showed the lowest (0.06). Correspondingly the highest specificity was shown by IPDS-2 (0.99) and the lowest by IPDS-1 and IPDS-10 (0.56). The highest positive predictive value was shown by IPDS-7 with 0.82 and the lowest was IPDS-3 with 0.03. Maximum negative predictive value was found for IPDS-2 (0.99) and minimum for IPDS-3 with 0.53. IPDS-5 had the highest proportion of PDs cases correctly classified (0.78) while the lowest proportion (0.52) was found for IPDS-2.

### IPDS item combinations

We tried out the screening properties of various IPDS item combinations. If all 11 items were used, a cut-off of ≥4 positive criteria seemed to have the best case-findings properties (Table 4). We found that the various shorter versions of the IPDS introduced by Langbehn et al. [12] had similar diagnostic properties as the full scale. We also introduced a new combination consisting of the five IPDS items that had a correct classification ≥0.66 (items #1, 4-6, 9), and found a cut-off ≥2 had good screening properties.

**Table 4:** Various IPDS combinations with their cut-off scores and their sensitivity, specificity, predictive value of positive test (PVP) and predictive value of negative test (PVN) as well as proportion of cases correctly classified

IPDS item combinations	Cut-off score	Sensitivity	Specificity	PVP	PVN	Correctly Classified
1 - 11	3	0.89	0.57	0.76	0.68	0.73
	4	0.77	0.71	0.73	0.75	0.74
	5	0.68	0.9	0.88	0.74	0.79
1 - 6	2	0.95	0.58	0.7	0.91	0.77
	3	0.69	0.81	0.78	0.72	0.74
	4	0.43	0.94	0.89	0.62	0.68
4 - 8	2	0.82	0.74	0.76	0.8	0.78
	3	0.62	0.9	0.87	0.7	0.76
	4	0.34	0.99	0.96	0.6	0.66
1, 3 - 8	2	0.96	0.53	0.67	0.93	0.74
	3	0.73	0.75	0.75	0.73	0.74
	4	0.59	0.92	0.88	0.69	0.75
1, 4-6, 9	2	0.93	0.6	0.7	0.9	0.77
	3	0.71	0.8	0.78	0.73	0.75
	4	0.48	0.94	0.9	0.64	0.71

The receiver operating analysis of the 11 items version of the IPDS showed an area under the curve of 0.86 for the IPDS in relation to PDs present or absent, and the optimal cut-off value of ≥4, showed a sensitivity of 0.77 and specificity of 0.71.

Among the shorter versions we mention good properties of the IPDS items 4-8 and cut-off ≥ 2 with sensitivity 0.82, specificity 0.74 and area under the curve of 0.84, since this version was used in the Oslo Health Survey [24].

### IPDS as a latent second order variable

We specified IPDS as a second order variable utilizing the PLSPM statistics, and the results are shown in Figure 1. As the figure shows, the measured variables loaded in general adequately on their respective first order variables. The exception from this is the item IPDS-2 with a weak (i.e. 0.27) and insignificant (i.e. t-value 1.69) factor loading. The remaining ten items had significant loadings (i.e. t-value > 1.96) associated with their respective first order variables. Four of five first order variables loaded in general adequately on their second order variable. Histrionic PD (HST) loaded only with 0.34 and we categorize this as a relatively weak loading. All five second order loadings had however t-values significantly > 1.96.

### IPDS in relation to the various PDs

The hit rates in relation to the PDs were examined with a cut-off level ≥4 of all 11 IPDS items (Table 2). The overall positive hit rate was 83% in relation to 95 PDs diagnoses made. The hit rate was best for PD-NOS (0.94) and cluster C disorders (0.83), but somewhat weaker for cluster A (0.76) and cluster B (0.77).

The second order IPDS variable was specified as an antecedent of PDs. As Figure 1 shows, the standardized regression coefficient is 0.64 and the second order IPDS variable explains 41% variation in PDs. Tenenhaus et al. [25] have suggested a global fit measure for PLSPM: *Goodness of Fit *(0 <*GoF  *< 1), defined as the geometric mean of the average communality and average R^2 ^(for endogenous constructs). Based on Cohen's [26] recommendation for evaluation of effect sizes, Wetzels et al. [23] recommend the following evaluation criteria for GoF values: small = 0.1, medium = 0.25, and large = 0.36. These values may serve as baseline values for validating the model specified in Figure 1. For the complete model, we obtained a *GoF *value of 0.53, which exceeds the cut-off value of 0.36 for large effect sizes of *R^2 ^*and allows us to conclude that our model performs well compared to the baseline values defined above.

## Discussion

In this study we observed: 1) No socio-demographic or psychological variables studied by us are confounding the IPDS as a screener for PDs. 2) The sensitivity and specificity of the IPDS supported the values reported by Germans et al. [15]. 3) The PLSPM analysis of the IPDS showed satisfactory coefficients (cf. standardized regression coefficient and factor loadings) and an adequate fit value.

We found that the GSI and the GAF-S as measures of psychopathology and the GAF-F as a measure of function as well as the IPDS were significantly associated with the presence of PDs in bivariate analysis. A new finding is that only the IPDS score remained significant in the multivariate analysis. Our interpretation of these results is that psychological and functional variables do not seem to interfere to any significant extent on the IPDS as a screener for PDs.

Among the previous studies of the screening properties of the IPDS, comparisons with the study of Germans et al. [15] is the most relevant one since they also studied psychiatric outpatients and had a base rate of 50%. Our findings concerning the IPDS on sensitivity, specificity, positive and negative predictive value, and proportion correctly classified were close to those of Germans et al., and could be considered as a replication. In POC samples with a base rate of 50% for PDs, a sensitivity of 0.82 a specificity of 0.74, seem to the optimal screening ability reached by the IPDS using a brief 5 items version consisting of the IPDS items 4-8 with cut-off ≥ 2 positive items.

What do such figures mean in practical clinical work? In a sample of 100 patients admitted to the POC, 50 have PDs, when the PDs base rate is 50%. A sensitivity of 0.82 tells that 41 (50 * 0.82) of these 50 PDs patients are correctly identified, while 9 are missed as false negatives. Among the 50 patients without PDs 37 (50 * 0.74) are correctly identified without PDs, while 13 are rated as false positive for PDs. Taken together 78 of the 100 patients are correctly classified. Doing 54 (41+13) instead of 100 SCID-II interviews, will miss 9 PDs patients and have 13 negative SCID-II interviews. If this consequence of sparing 46 interviews is considered suboptimal, setting a lower cut-off with higher sensitivity will reduce the number of PDs patients missed, however at a price of performing more negative SCID-II interviews. Therefore the cut-off value of the items, as well as the item combination used should be considered when the price of false negatives and false positives are considered at the local POC.

The PLSPM analysis indicated a relatively strong relation between the IPDS and PDs, i.e. IPDS explains 41 percent of the variation in the PDs. The analysis also supports IPDS as a second order construct with five different sub dimensions. Both a set of satisfactory factor loadings and an adequate fit value support this conceptualization of IPDS. Two factor loadings were, however, relatively weak; cf. the concept histrionic PD in Figure 1 and the low coefficients of 0.27 and 0.34. This may indicate that histrionic PD does not represent a valid dimension of IPDS, but it may as well be a result of setting specific conditions. Our sample was relatively low (N = 145) and it is legitimate to ask if this is large enough for the second order PLSPM analysis. PLSPM is categorized as a "soft modeling technique" if compared with covariance based structure equation modeling technique (such as LISREL). Soft modeling means an approach where no strong assumptions (with respect to the distributions, the sample size and the measurement scale) are required [27], and we therefore conclude that our sample size is adequate for the second order PLSPM analysis. However, further research is clearly needed to address these issues.

The positive hit rate of the 11 item version of the IPDS with cut-off ≥4 varied from 76% for cluster A PDs to 94% for PD-NOS (Table 2). These findings were in accordance with those of Germans et al. [15]. When we compared the distribution of positive ratings of the 11 IPDS items, item #5 (social anxiety) and item #6 (unwilling to get involved) were significantly more common in our sample than in Germans et al., while the distribution of the other 9 items did not differ significantly. The most probable explanation is differences in the diagnostic distribution of the samples, since our sample contained significantly more cluster A and C PDs and significantly fewer cluster B PDs compared to the sample of Germans et al.

We also want to point out the considerable difference between the IPDS items concerning their proportions of correct classification. The two best items (item #5 and #6, with 78% and 74%, respectively) belonged to avoidant PD, while the two poorest ones (#2 and #3 with 52% and 55%, respectively) belonged to histrionic PD. This result confirms the finding from the path analysis, namely that the histrionic items are the weakest ones in relation to the PD concept of the IPDS.

Our results have to be considered in the light of some limitations. The reference diagnoses based on the SCID-II interviews were performed by 22 therapists, that each did from 1 to 15 interviews. In spite of the SCID-II training seminar, there is a definite risk for heterogeneity of the diagnostic practice concerning PDs. Further, we included 145 patients, which could be considered as suboptimal for the power of some of the statistical tests. The exclusion of patients referred with drug and alcohol dependence as main diagnosis might contribute to a selection bias, mostly decreasing the prevalence rate of cluster B PDs. A certain degree of consensus has emerged concerning prevalence rates of PDs in the general population [4,28]. Seeking treatment is however related to a number of clinical and demographical factors [29], and prevalence rates and distribution of PDs in clinical samples in vary considerably with methodological and diagnostic tools used in the assessments [1]. In The Rhode Island Methods to Improve Diagnostic Assessment and Services (MIDAS) project [30] patients referred to a community based POC were diagnosed with reliable and valid procedures. The project found a base rate of 45% for PDs and a 24% prevalence rate Cluster B among those having a PD. Despite our lower prevalence rate of 14% and Germans et al. [15] higher prevalence rate of 48% of Cluster B the sensitivity and specificity of IPDS in the studies are fairly comparable.

Finally, the IPDS was developed using 11 DSM-III-R criteria for PDs. 10 of these criteria were retained in DSM-IV, and one (histrionic PD criterion 7) was omitted. This omission is a minor point in our view since we test to what extent a set of criteria function as a good screening for PDs in DSM-IV. Such a task does demand that the criteria are derived from DSM-IV, although that would have been to some advantage.

Since performing SCID-II interviews are extensive time consuming a screening instrument for PDs is needed in POC due to heavy work burdens and lack of qualified SCID-II interviewers. Taking the limitations of the study into account we regard the short and feasible IPDS in Norwegian as a useful screening instrument in a busy clinical setting until the revision of the DSM-IV is completed.

## Conclusions

In conclusion, our results give support to the IPDS as a useful screening instrument for PDs present or absent in the POC setting. Particularly, several of the shorter versions seem to have better case finding abilities than the full version of the IPDS.

## Competing interests

The authors declare that they have no competing interests.

## Authors' contributions

IO participated in the design, collected data and drafted the manuscript of the study. ØS performed statistical analyses and helped to draft the manuscript. AAD participated in the design, performed statistical analyses and helped to draft the manuscript of the study. All authors have read and approved the final manuscript

## Pre-publication history

The pre-publication history for this paper can be accessed here:

http://www.biomedcentral.com/1471-244X/11/105/prepub
